# Risk factors for pressure injury development in critically ill patients in the intensive care unit: a systematic review protocol

**DOI:** 10.1186/s13643-017-0451-5

**Published:** 2017-03-20

**Authors:** Fiona Coyer, Nahla Tayyib

**Affiliations:** 1 School of Nursing, Queensland University of Technology, Victoria Park Rd, Kelvin Grove, Queensland 4059 Australia; 2 Intensive Care Services, Royal Brisbane and Women’s Hospital, Butterfield St, Herston, Queensland 4006 Australia; 3 School of Nursing, Umm Al-Qura University, Mecca, Saudi Arabia; 4 Institute for Skin Integrity and Infection Prevention, University of Huddersfield, Huddersfield, UK

**Keywords:** Intensive/critical care, Pressure injury, Pressure ulcer, Predictors, Risk factors

## Abstract

**Background:**

Pressure injuries (PIs) create a significant burden in the health care system. Up to 49% of critically ill patients develop PIs. Identifying and understanding potential risk factors is essential to the provision of effective targeted prevention strategies to mitigate risk. The objectives of this review are to identify patient-centred clinical factors that may be associated with PI development in the adult intensive care environment and to determine the effect size of the relationship between identified factors and PI development in this unique population.

**Method/design:**

The review will follow the PRISMA reporting guidelines for systematic reviews. Electronic databases (Cochrane; PubMed/MEDLINE; CINAHL (EBSCOhost); Embase; Scopus; PsycINFO; Proquest; Networked Digital Library of Theses and Dissertations; Australian Digital Theses Program, Grey literature, Google scholar, and Clinical Trial Registries) will be systematically searched. A suite of search terms will identify articles that have examined the patient-centred risk factors for PI development in adult intensive care units. The search strategy will be designed to retrieve studies published since inception to 2016 in English language. Quality of the studies will be assessed by using an assessment framework designed to appraise quality in prognostic studies and methodological considerations in the analysis and publication of observational studies. Screening, study selection process, and data extraction will be undertaken by two independent reviewers. Disagreement will be resolved by discussion and, if required, a third independent reviewer. Clinical and methodological heterogeneity across studies will be assessed and, if possible, meta-analyses will be performed.

**Discussion:**

The evidence synthesis arising from this review will identify person-centred risk factors that are associated with PI development among critically ill patients in intensive care. Findings from this review will demonstrate potential patient risk factors that may influence practice and research priorities to prevent PI development and improve the quality of care provided.

**Systematic review registration:**

PROSPERO CRD42016037690

**Electronic supplementary material:**

The online version of this article (doi:10.1186/s13643-017-0451-5) contains supplementary material, which is available to authorized users.

## Protocol

### Background

Pressure injuries (PIs) represent a common but potentially preventable condition seen most often in high-risk populations such as elderly persons, those with physical impairments, and the critically ill [1–3]. Pressure injuries, also known as pressure ulcers, are defined as a damage or lesion to the skin and underlying soft tissue, resulting from unrelieved pressure, shear, friction, moisture, or a combination of these, usually over a bony prominence or an anatomical area related to medical devices [4]. Pressure injuries differ in size and in the severity of affected tissue layers, with the latter ranging from skin erythema to muscle and underlying bone damage [4]. Moreover, PIs have significant negative impacts related to patients, society, and health systems (e.g. pain, increased infection rates, morbidity and mortality, increased length of stay in hospital, and raised financial costs) [5–7]. Preventing PI development to reduce the burden of PIs for patients and health care systems is considered a core aim of healthcare organizations.

Evidence suggests that PIs can be prevented with the implementation of PI prevention guidelines or care bundles, which target known risk factors associated with PI development [8–10]. Therefore, the identification and understanding of risk factors is required in order to provide appropriate prevention interventions and better utilize resources in practice.

Initial searching identified a systematic review published on the risk factors associated with PI development in adult hospitalized patients [11]. Findings identified that the three main factors which contributed to PI development were reduced mobility/activity, perfusion alterations (e.g. diabetes, vascular disease, poor circulation, blood pressure changes, smoking, oedema), and skin or PI status (e.g. a history of a previous PI occurrence). Further, the review concluded that PI occurrences cannot be explained by a single factor. This significant systematic review reported a total of 54 studies of which 13 studies were conducted in the intensive care environment [11]. However, the review reported only aggregate data from the 54 studies and no analyses were reported on specific subpopulations such as critically ill intensive care unit (ICU) patients. In comparison to general adult acute care in-patients, critically ill patients are more susceptible to risk factors for PI development as the majority of critically ill patients are ventilated and sedated and, therefore, unable to care for themselves, move, or change position. Further, the patient’s critical illness may involve hemodynamic instability and oxygenation disorders, which potentially may complicate and accelerate the effects of prolonged immobility such as PI development. Extensive exposure to pressure, from lying or sitting, on a specific part of the body renders patients at greater risk of skin breakdown. The highest PI in-hospital prevalence and incidence rates are noted in critically ill patients, thus confirming that, when compared to the total hospital population, the critically ill are at greatest risk for PI development [12]. It is estimated that up to 40% of patients develop PIs during their admission to ICUs [13].

It has been argued that PI development is a complex phenomenon that is enhanced by the presence of multiple, rather than single, risk factors in the individual [14]. Two literature reviews have been retrieved that have addressed the risk factors of PI development in the intensive care context [15, 16]. Findings from the first review [15] revealed that the potential risk factors for PI development were the same among hospitalized patients, despite critically ill patients having more than one factor. These findings were supported with a second review [16], which additionally explored different risk factors that accelerate the development of PI in ICU contexts and have an influence on the level and extent of tissue necrosis. These factors were identified and conceptualized in two categories: intrinsic (inherent factors of critical illness) and extrinsic (related to external forces) factors. A total of 28 factors were identified as the main risk factors for PI development in ICU settings in two or more studies. The intrinsic factors identified in two or more studies were older age, increased length of stay in the ICU, and history of cardiovascular disease. The extrinsic factors identified in two or more studies were the administration of norepinephrine and patient repositioning (turning). However, these were literature reviews [15, 16] in which no assessment of the methodological quality of the studies reviewed was undertaken thus making the interpretation of findings more susceptible to bias. In addition, Coleman and colleagues [11] suggest in their systematic review that multivariable statistical modelling, the identification of potential factors associated with PI development, should be independent to other risk factor variables included. An independent risk factor does not infer causality. Rather, it is a risk factor that ‘retains statistical association with the outcome when other established risk factors are included in the statistical model’ [17]. Thus, truly independent predictive intrinsic, patient-centred risk factors for critically ill patients in the ICU have yet to be conclusively established.

A systematic review is required to synthesize comprehensive current evidence in order to identify potential independent patient-centred clinical factors that are associated with PI development among critically ill patients in intensive care. Currently, after searching across PubMed, CINAHL, the Cochrane Library, the Joanna Briggs Institute Database of Systematic Reviews and Implementation Reports, and Google Scholar, no current systematic review addressing risk factors of PIs in this context has been identified. Identifying the potential independent person or patient-centred factors will facilitate decision-makers such as researchers, clinicians, and policy-makers to provide appropriate interventions to alleviate the pressing problem of PI development.

## Objectives

The objectives of this review are to (1) identify patient-centred clinical factors that may be associated with PI development in adult intensive care and (2) determine the effect size of the association between identified factors and PI development. The systematic review will synthesize existing knowledge and make recommendations for future research.

## Methods/design

This review will replicate the methodology used by Coleman and colleagues who identified PI risk factors in adult hospitalized patients [11]. However, this review will focus on adult intensive care patients.

This protocol adheres to the Preferred Reporting Items for Systematic Reviews and Meta-Analyses Protocol (PRISMA-P) statement [18], and this is provided as an Additional file 1. This review is registered with PROSPERO—registration number CRD42016037690.

### Criteria for considering studies for this review

#### Types of studies

Eligible quantitative studies will be included in this review. The systematic review will consist of studies using either epidemiological designs such as retrospective or prospective cohort studies, or experimental designs such as randomized controlled trials or quasi-experimental trials, where analyses for potential patient-centred clinical factors of PI development in intensive care have been reported. Cross-sectional, qualitative, case studies and abstract-only reports, for which full text is not available, will be excluded from this review.

#### Type of clinical settings

Studies conducted in intensive/critical care settings will be included.

#### Type of participants

This review will consider studies that included adult critically ill patients aged 18 or above, who have not been diagnosed with a PI that developed before the patient was admitted to the ICU.

#### Type of exposure

No restriction will be imposed for risk factors that are significantly associated with PI development in intensive care. Patient risk factors are defined as those factors related to individual patient descriptors including age, gender, diagnosis, comorbidities, body mass index, severity of illness, treatment modalities related to critical illness, complications of treatment, clinical pathology, length of ICU stay, and ICU outcome (discharge, transfer, or death) [16]. In addition, studies that examine the factors of PI development for adults across hospitals but have separate statistical analyses reported for PI development in adult intensive care will also be included.

#### Type of outcome measure

This review will consider studies that include all stages of PI or equivalent as the outcome measure. According to the National Pressure Ulcer Advisory Panel, European Pressure Ulcer Advisory Panel, and Pan Pacific Pressure Injury Alliance Clinical practice guidelines, the PI stages reflect the level of skin tissue damage [4, 19].

### Search methods of identification of studies

The search strategy aims to find both published and unpublished studies exploring the risk factors that are associated with PI development in intensive care. The literature will be searched using Medical Subject Headings (MeSH) and combinations of key terms. The search is limited to English language publications with no restriction on the year of publication. The electronic databases searches will include the Cochrane database (1991 (established) to present); PubMed/MEDLINE (1969–present); CINAHL (EBSCOhost) (1989 to present); Embase (1966–present); Scopus (1975–present); PsycINFO (2009 to present); Proquest (1959 to present); and Google scholar. In addition, the reference lists of articles meeting the criteria for this review will be searched for further relevant studies. The search for unpublished studies will include Networked Digital Library of Theses and Dissertations; Australian Digital Theses Program; Open Grey (greynet.org); science.gov; clinicaltrials.gov; International Standards for Randomised Controlled Trials (IRSCTN) Registry; and the Australian and New Zealand Clinical Trials Registry (ANZCTR).

The search syntax will be designed and guided by the Cochrane Handbook [20] to retrieve the relevant results. Search strings will be focused on the terms pressure ulcer, pressure injury, risk factors, predictors, adult intensive care, and their synonyms.

### Search terms

Medical Subject Headings (MeSH) and free text word terms will be developed specifically for each database and will be subsequently pilot tested. For example, for MeSH, terms include (pressure ulcer* OR pressure injur* OR bed sore* OR decubitus ulcer* OR pressure sore*) AND (risk factor* OR predictor* OR predictor factor*) AND (intensive care* OR ICU OR critical care) AND (adult OR age ≥18).

### Data collection and analysis

#### Selection of studies

EndNote (Thomson Reuters) software will be used to manage records for retrieval of articles and inclusion/exclusion decisions. Two independent reviewers (both authors) will perform the initial screening of titles and abstracts to identify eligible studies (Additional file 2). Full copies of all studies that meet the criteria as outlined below will be obtained for further assessment. The inclusion criteria are (i) length of patient follow-up that is at least 48 h (this has been identified as an accepted time frame for PI development) [21], (ii) multivariate analyses undertaken to identify factors affecting PI outcome, and (iii) not specific to geographical areas. The exclusion criteria are (i) observational studies that will be excluded if >20% of the study sample were excluded from analysis for reasons including withdrawal, death, loss to follow-up, and missing records and (ii) control trial studies will be excluded unless all of the following minimum criteria are applied: parts of the study were prospectively designed and intention to treat analyses are reported [20].

### Data extraction and management

Data will be extracted independently by the two reviewers using the data extraction form. This form will include the study characteristics (for example, study population, recruitment type used, PI definition, and analysis method) and risk factors investigated in the multivariate models, including those found to be significant. Non-significant factors through using a stepwise regression will be included if it reported as independent correlated variables to PI development. Disagreement will be reconciled by discussion leading to mutual agreement or, if this is not possible, consultation with a third independent reviewer. In addition, authors will be contacted if papers are unobtainable or the methods reported need clarification.

### Assessment of risk of bias in included studies

Both authors will undertake the risk of bias assessment of the included studies independently, as guided by the assessment framework for assessing quality in prognostic studies and methodological considerations in the analysis and publication of observational studies [11] as described below.

### Quality assessment

The methodological quality of eligible studies will be assessed and critically appraised by two independent reviewers (both authors) using the assessment framework that is designed to appraise quality in prognostic studies and methodological considerations in the analysis and publication of observational studies [11]. Any disagreements that arise between the reviewers will be resolved through discussion or, if agreement is not reached, consultation with a third, independent reviewer.

The quality assessment tool will rate the relevant methodological parameters of studies across seven areas [11]: selection bias (selection of target population), confounders (whether confounders were controlled using appropriate adjustment), data collection methods (validity and reliability by using a clear definition or description of the risk factor), reporting of continuous variables (cut point), measurement of the range of potential risk factors, withdrawals and dropouts (dropout rates and completion of study rates), and no selective reporting of results.

In addition, this tool has four considerations to appraise domains quality:

1. Is there a sufficient number of events (rule of thumb, 10 events per risk factor)?
2. Is there sufficient presentation of data to assess the adequacy of method and analysis?
3. Is the strategy for model building (i.e. inclusion of variables) appropriate and based upon a conceptual framework?
4. Is the selected model adequate for the design?

Each criteria will be assessed as being met (yes/no/partial/unsure). Consequently, assessment of domain criteria will assist the determination of overall study quality [11].

#### Classification of study quality

The quality of the studies will be classified according to the assessment framework for assessing quality and methodological consideration in the analysis, meta-analysis, and publication of observational studies [11] as high, moderate, low, and very low quality based on the following criteria:

- High: yes for all four criteria
- Moderate: yes for criteria 1 and at least 2 for other criteria
- Low: no for criteria 1 and no or partial for 2 other criteria
- Very low: no for criteria 1 and no or partial for all 3 other criteria

#### Unit/scale of analysis issues

The unit of analysis will be based on the individual patient.

#### Assessment of heterogeneity

The studies will be assessed by considering variability in the clinical and methodological heterogeneity to ensure sufficient homogeneity and rigour of the studies to conduct the meta-analysis procedure. Clinical heterogeneity of the studies will be assessed by considering their population, risk factor description, and outcome. Methodological heterogeneity will be assessed using the data extracted on study design, procedure, and analysis reported. In the absence of clinical and methodological heterogeneity statistical measures, *I* ^2^ and H^2^_M_ statistic measures [22, 23] will also be used to determine the level of consistency between studies. *I* ^2^ and H^2^_M_ values will be used to measure the impact of heterogeneity between and within the study variance. An *I* ^2^ ≥ 50%, which corresponds to H^2^_M_ > 0, will be considered a significant level of heterogeneity.

#### Assessment of publication bias

The risk of publication bias will be minimized by comprehensively searching databases and obtaining data from unpublished work to reduce the risk of reporting bias [24, 25]. Moreover, if more than 10 studies are reviewed that fit the criteria, a funnel plot will be used to determine the relationship between study size and effect power of cohort clinical trial studies by signs of asymmetry.

#### Data synthesis

A meta-analysis may not be able to be conducted due to the clinical or statistical heterogeneity of the eligible studies. However, if included studies are sufficiently homogenous and rigorous, a meta-analysis using a random-effects model will be conducted. A relative risk will be the appropriate indicator of effect for potential correlates in this review with a 95% confidence interval (CI) calculated for binary outcomes (PI development). However, if there is considerable clinical and design heterogeneity in the included studies, the findings will be presented in narrative form including tables to aid in data presentation where appropriate.

## Summary of findings

Patient-centred clinical risk factors for PI development will be categorized by domains and subdomains according to Coleman and colleagues [11]. Coding contributory factors into different domains will be conducted by the two authors independently. The identified domain and sub-domain factor will be summarized and presented in tables. Each sub-domain factor will be summarized including a number of significant studies using a multivariable analysis and the total number and quality of the studies entering the variable. Disagreements will be resolved by discussion to achieve consensus or, if this is not possible, by a third independent reviewer.

## Additional files

Additional file 1: PRISMA-P (Preferred Reporting Items for Systematic Review and Meta-Analysis Protocols) 2015 checklist. (PDF 278 kb)
Additional file 2: Figure S1. Literature searches, screening and selection of articles for inclusion. (PDF 102 kb)

## Abbreviations

CI: Confidence interval
ICU: Intensive care unit
PI: Pressure injury

## References

[1] Gedamu H, Hailu M, Amano A (2014). Prevalence and associated factors of pressure ulcers among hospitalized patients at Felegehiwot referral hospital, Bahir Dar, Ethiopia. Adv Nurs.

[2] Cox J (2011). Predictors of pressure ulcers in adult critical care patients. Am J Crit Care.

[3] Reddy M, Gill SS, Rochon PA (2006). Preventing pressure ulcers: a systematic review. JAMA.

[4] National Pressure Ulcer Advisory Panel (NPUAP). 2016. Announcement of change in terminology from pressure ulcer to pressure injury. http://www.npuap.org/national-pressure-ulcer-advisory-panel-npuap-announces-a-change-in-terminology-from-pressure-ulcer-to-pressure-injury-and-updates-the-stages-of-pressure-injury/.

[5] Bennett G, Dealey C, Posnett J (2004). The cost of pressure ulcers in the UK. Age Ageing.

[6] Graves N, Birrell F, Whitby M (2005). Effect of pressure ulcers on length of hospital stay. Infect Control Hosp Epidemiol.

[7] Pieper B, Langemo D, Cuddigan J (2009). Pressure ulcer pain: a systematic literature review and National Pressure Ulcer Advisory Panel white paper. Ostomy Wound Manag.

[8] Elliott R, McKinley S, Fox V (2008). Quality improvement program to reduce the prevalence of pressure ulcers in an intensive care unit. Am J Crit Care.

[9] Gray-Siracusa K, Schrier L (2011). Use of an intervention bundle to eliminate pressure ulcers in critical care. J Nurs Care Qual.

[10] Schindler CA. More than S.K.I.N. Deep: decreasing pressure ulcer development in the pediatric intensive care unit. Marquette University; 2009. http://epublications.marquette.edu/dissertations_mu/85/.

[11] Coleman S, Gorecki C, Nelson EA, Closs SJ, Defloor T, Halfens R, Farrin A, Brown J, Schoonhoven L, Nixon J (2013). Patient risk factors for pressure ulcer development: systematic review. Int J Nurs Stud.

[12] Berlowitz D, Thomas D, Compton M (2014). Incidence and prevalence of pressure ulcers. Pressure ulcers in the aging population: a guide for clinicians.

[13] Tayyib N, Coyer F, Lewis P (2016). Saudi Arabian adult intensive care unit pressure ulcer incidence and risk factors: a prospective cohort study. Int Wound J.

[14] Shahin ES, Dassen T, Halfens RJ (2009). Incidence, prevention and treatment of pressure ulcers in intensive care patients: a longitudinal study. Int J Nurs Stud.

[15] Keller PB, Wille J, van Ramshorst B, van der Werken C (2002). Pressure ulcers in intensive care patients: a review of risks and prevention. Intensive Care Med.

[16] Tayyib N, Coyer F, Lewis P (2013). Pressure ulcers in the adult intensive care unit: a literature review of patient risk factors and risk assessment scales. J Nurs Educ Pract.

[17] Brotman DJ, Walker E, Lauer MS, O’Brien OG (2005). In search of fewer independent risk factors. Arch Intern Med.

[18] Moher D, Liberati A, Tetzlaff J, Altman DG, PRISMA Group (2009). Preferred reporting items for systematic reviews and meta-analyses: the PRISMA statement. BMJ.

[19] Emily H, National Pressure Ulcer Advisory Panel, European Pressure Ulcer Advisory Panel, & Pan Pacific Pressure Injury Alliance (2014). Prevention and treatment of pressure ulcers: quick reference guide.

[20] Higgins JPT, Green S (editors). Cochrane Handbook for Systematic Reviews of interventions. Version 5.1.0 [updated March 2011]. The Cochrane Collaboration, 2011. Available from www.handbook.cochrane.org.

[21] Black JM, Brindle CT, Honaker JS (2016). Differential diagnosis of suspected deep tissue injury. Int Wound J.

[22] Huedo-Medina TB, Sánchez-Meca J, Marín-Martínez F, Botella J (2006). Assessing heterogeneity in meta-analysis: Q statistic or I^2^ index?. Psychol Methods.

[23] Mittlböck M, Heinzl H (2006). A simulation study comparing properties of heterogeneity measures in meta‐analyses. Stat Med.

[24] Glasziou P, Ebrary I (2001). Systematic reviews in health care: a practical guide.

[25] Song F, Hooper L, Loke YK (2013). Publication bias: what is it? How do we measure it? How do we avoid it?. Open Access J Clin Trials.

