# Supplementary material for: Risk factors for pressure injury development in critically ill patients in the intensive care unit: a systematic review protocol
Source: Syst Rev. 2017 Mar 20;6:58. doi: 10.1186/s13643-017-0451-5 (PMC5359849; doi:10.1186/s13643-017-0451-5)
Supplement: Additional file 1: — PRISMA-P (Preferred Reporting Items for Systematic Review and Meta-Analysis Protocols) 2015 checklist. (PDF 278 kb) [file 13643_2017_451_MOESM1_ESM.pdf]

PRISMA-P (Preferred Reporting Items for Systematic review and Meta-Analysis Protocols) 2015 checklist: recommended items to address in a systematic review protocol*

Section and topic	Item No	Checklist item
**ADMINISTRATIVE INFORMATION**		
Title:		Risk factors for pressure injury development in critically ill patients in the intensive care unit: a systematic review protocol.
Identification	1a	The report is a protocol of a systematic review
Update	1b	No
Registration	2	This review is registered with PROSPERO – registration number CRD42016037690.
Authors:		
Contact	3a	Fiona Coyer, MScNsg, PGCEA, PhD, RN (corresponding author)<br>Professor of Nursing,<br>School of Nursing, Queensland University of Technology and Intensive Care Services, Royal Brisbane and Women's Hospital, Brisbane, Australia.<br>Visiting Professor Institute for Skin Integrity and Infection Prevention, University of Huddersfield, United Kingdom.<br><br>Postal address: Intensive Care Services, Royal Brisbane & Women's Hospital, Level 3 Ned Hanlon Building, Butterfield St, Herston, Queensland, Australia, 4006<br>Tel: +61 7 36462140<br>Email: f.coyer@qut.edu.au<br><br>Nahla Tayyib, BN, MN, PhD, RN<br>School of Nursing, Umm Al-Qura University, Saudi Arabia<br>Tel: +61 459173020<br>Email: nahla_t@hotmail.com
Contributions	3b	FC and NT contributed equally to this work. Fc and NT will select the studies, extract the data, assess the risk of bias, and design the study. FC and NT contributed to the further writing of the manuscript as well as read and approved the final manuscript.
Amendments	4	This is a new protocol.
Support:		
Sources	5a	This review is not supported financially
Sponsor	5b	Not applicable
Role of sponsor or funder	5c	Not applicable

**INTRODUCTION**		
Rationale	6	Pressure injuries (PIs) represent a common but potentially preventable condition seen most often in high-risk populations such as elderly persons, those with physical impairments, and the critically ill [1-3]. Pressure injuries, also known as pressure ulcers, are defined as a damage or lesion to the skin and underlying soft tissue, resulting from unrelieved pressure, shear, friction, moisture, or a combination of these, usually over a bony prominence or an anatomical area related to medical devices [4]. Pressure injuries differ in size and in the severity of affected tissue layers, with the latter ranging from skin erythema to muscle and underlying bone damage [4]. Moreover, PIs have significant negative impacts related to patients, society, and health systems (e.g. pain, increased infection rates, morbidity and mortality, increased length of stay in hospital, and raised financial costs) [5-7]. Preventing PI development to reduce the burden of PIs for patients and health care systems is considered a core aim of healthcare organizations.<br>Evidence suggests that PIs can be prevented with the implementation of PI prevention guidelines or care bundles, which target known risk factors associated with PI development [8-10]. Therefore, the identification and understanding of risk factors is required in order to provide appropriate prevention interventions and better utilize resources in practice.<br>Initial searching identified a systematic review published on the risk factors associated with PI development in adult hospitalized patients [11]. Findings identified that the three main factors which contributed to PI development were reduced mobility/activity, perfusion alterations (e.g. diabetes, vascular disease, poor circulation, blood pressure changes, smoking, edema), and skin or PI status (e.g. a history of a previous PI occurrence). Further, the review concluded that PI occurrences cannot be explained by a single factor. This significant systematic review reported a total 54 studies of which 13 studies were conducted in the intensive care environment [11]. However, the review reported only aggregate data from the 54 studies and no analyses were reported on specific subpopulations such as critically ill intensive care unit (ICU) patients. In comparison to general adult acute care in-patients, critically ill patients are more susceptible to risk factors for PI development as the majority of critically ill patients are ventilated and sedated and, therefore, unable to care for themselves, move or change position. Further, the patient's critical illness may involve hemodynamic instability and oxygenation disorders, which potentially may complicate and accelerate the effects of prolonged immobility such as PI development. Extensive exposure to pressure, from lying or sitting, on a specific part of the body renders patients at greater risk of skin breakdown. The highest PI in-hospital prevalence and incidence rates are noted in critically ill patients, thus confirming that, when compared to the total hospital population the critically ill are at greatest risk for PI development [12]. It is estimated up to 40% of patients develop PIs during their admission to ICUs [13].<br>It has been argued that PI development is a complex phenomenon that is enhanced by the presence of multiple, rather than single, risk factors in the one individual [14]. Two literature reviews have been retrieved that have addressed the risk factors of PI development in the intensive care context [15, 16]. Findings from the first review [15] revealed that the potential risk factors for PI development were the same among hospitalized patients, despite critically ill patients having more than one factor. These findings were supported with a second review [16], which additionally explored different risk factors that accelerate the development of PI in ICU contexts and have an influence on the level and extent of tissue necrosis. These factors were identified and conceptualized in two categories: intrinsic (inherent factors of critical illness) and extrinsic (related to external forces) factors. A total of 28 factors were identified as the main risk factors for PI development in ICU settings in two or more studies. The intrinsic factors identified in two or more studies were older age, increased length of stay in the ICU, and history of cardiovascular disease. The extrinsic factors identified in two or more studies were the administration of norepinephrine and patient repositioning (turning). However, these were literature reviews [15,16] in which no assessment of the methodological quality of the studies reviewed was undertaken thus making the interpretation of findings more susceptible to bias. In addition, Coleman and colleagues [11] suggest in their systematic review that multivariable statistical modelling, the identification of potential factors associated with PI development, should be independent to other risk factor variables included. An independent risk factor does not infer causality.

		Rather it is a risk factor that 'retains statistical association with the outcome when other established risk factors are included in the statistical model'[17]. Thus, truly independent predictive intrinsic, patient centered risk factors for critically ill patients in the ICU have yet to be conclusively established.<br>A systematic review is required to synthesize comprehensive current evidence in order to identify potential independent patient centred clinical factors that are associated with PI development among critically ill patients in intensive care. Currently, after searching across PubMed, CINAHL, the Cochrane Library, the Joanna Briggs Institute Database of Systematic Reviews and Implementation Reports and Google Scholar, no current systematic review addressing risk factors of PIs in this context has been identified. Identifying the potential independent person or patient-centred factors will facilitate decision-makers such as researchers, clinicians, and policy-makers to provide appropriate interventions to alleviate the pressing problem of PI development.
Objectives	7	The objectives of this review are to 1) identify patient centered clinical factors that may be associated with PI development in adult intensive care and 2) determine the effect size of the association between identified factors and PI development. The systematic review will synthesize existing knowledge and make recommendations for future research.
**METHODS**		
Eligibility criteria	8	**Criteria for considering studies for this review**<br>- **Types of studies**<br>Eligible quantitative studies will be included in this review. The systematic review will consist of studies using either epidemiological designs such as retrospective or prospective cohort studies, or experimental designs such as randomized controlled trials or quasi-experimental trials, where analyses for potential patient centered clinical factors of PI development in intensive care have been reported. Cross-sectional, qualitative, case studies and abstract-only reports, for which full text is not available, will be excluded from this review.<br>- **Type of clinical settings**<br>Studies conducted in intensive/critical care settings will be included.<br>- **Type of participants**<br>This review will consider studies that included adult critically ill patients aged 18 or above, who have not been diagnosed with a PI that developed before the patient was admitted to the ICU.<br>- **Type of exposure:**<br>No restriction will be imposed for risk factors that are significantly associated with PI development in intensive care. Patient risk factors are defined as those factors related to individual patient descriptors including age, gender, diagnosis, comorbidities, body mass index, severity of illness, treatment modalities related to critical illness, complications of treatment, clinical pathology, length of ICU stay and ICU outcome (discharge, transfer or death) [16]. In addition, studies that examine the factors of PI development for adults across hospitals but have separate statistical analyses reported for PI development in adult intensive care will also be included.<br>- **Type of outcome measure**<br>This review will consider studies that include all stages of PI or equivalent as the outcome measure. According to the National Pressure Ulcer Advisory Panel, European Pressure Ulcer Advisory Panel and Pan Pacific Pressure Injury Alliance Clinical practice guidelines the PI stages reflect the level of skin tissue damage [4, 19].

Information sources	9	The search strategy aims to find both published and unpublished studies exploring the risk factors that are associated with PI development in intensive care. The literature will be searched using Medical Subject Headings (MeSH) and combinations of key terms. The search will be limited to English language publications with no restriction on the year of publication. The electronic databases searches will include: the Cochrane (1991 (established) to present); PubMed/MEDLINE (1969–present); CINAHL (EBSCOhost) (1989 to present); Embase (1966–present); Scopus (1975–present); PsycINFO (2009 to present); Proquest (1959 to present); and Google Scholar. In addition, the reference lists of articles meeting the criteria for this review will be searched for further relevant studies. The search for unpublished studies will include: Networked Digital Library of Theses and Dissertations; Australian Digital Theses Program; Open Grey (greynet.org); science.gov; clinicaltrials.gov; International Standards for Randomised Controlled Trials (IRSCTN) Registry; and the Australian and New Zealand Clinical Trials Registry (ANZCTR).
Search strategy	10	The search syntax will be designed by applying the guidance of the Cochrane Handbook [18] to retrieve the relevant results. Search strings will be focused on the terms pressure ulcer, pressure injury, risk factors, predictors, adult intensive care, and their synonyms.
Study records:		
Data management	11a	FC and NT will independently extract data using the data extraction form (Additional file 1). Any differences of opinion will be reconciled by mutual agreement. Data will be entered into a database (Review Manager (RevMan), Version 5) for statistical analysis.
Selection process	11b	EndNote (Thomson Reuters) software will used to manage records for retrieval articles and inclusion/exclusion decisions. Two independent reviewers (both authors) will perform the initial screening of titles and abstracts to identify eligible studies. Full copies of all studies that meet the criteria as outlined below will be obtained for further assessment. The inclusion criteria are (i) length of patient follow-up is at least 48 hours (this has been identified as an accepted time frame for PI development) [21] (ii) multivariate analyses undertaken to identify factors affecting PI outcome and (iii) not specific to geographical areas. The exclusion criteria are (i) observational studies will be excluded if >20% of the study sample were excluded from analysis for reasons including withdrawal, death, loss to follow-up and missing records (ii) control trial studies will be excluded unless all of the following minimum criteria applied: parts of the study were prospectively designed and intention to treat analyses are reported [20]. More detail will be illustrated on a PRISMA flow diagram for included and excluded studies during a selection process.
Data collection process	11c	Data will be extracted independently by the two reviewers using the data extraction form. This form will include: the study characteristics (for example, study population, recruitment type used, PI definition and analysis method) and risk factors investigated in the multivariate models, including those found to be significant. Non-significant factors through using a stepwise regression will be included if it reported as independent correlated variables to PI development. Disagreement will be reconciled by discussion leading to mutual agreement or if this is not possible, consultation with a third independent reviewer. In addition, authors will be contacted if papers are unobtainable or the methods reported needs clarification
Data items	12	Variables are individual patient descriptors including age, gender, diagnosis, comorbidities, body mass index, severity of illness, treatment modalities related to critical illness, complications of treatment, clinical pathology, length of ICU stay and ICU outcome (discharge, transfer or death).

Outcomes and prioritization	13	This review will consider studies that include all stages of PI or equivalent as the outcome measure. According to the National Pressure Ulcer Advisory Panel, European Pressure Ulcer Advisory Panel and Pan Pacific Pressure Injury Alliance Clinical practice guidelines the PI stages reflect the level of skin tissue damage [4, 17].
Risk of bias in individual studies	14	FC and NT will undertake the risk of bias assessment of the included studies independently, as guided by the assessment framework for assessing quality in prognostic studies and methodological considerations in the analysis and publication of observational studies [11] as described below.<br>**Quality assessment**<br>The methodological quality of eligible studies will be assessed and critically appraised by two independent reviewers (FC & NT) using the assessment framework that is designed to appraise quality in prognostic studies and methodological considerations in the analysis and publication of observational studies [11]. Any disagreements that arise between the reviewers will be resolved through discussion, or with a third reviewer.<br>The quality assessment tool will rate the relevant methodological parameters of studies across seven areas: selection bias (selection of target population), confounders (whether confounders were controlled using appropriate adjustment), data collection methods (validity and reliability by using a clear definition or description of the risk factor), continuous variable are reported (cut point), range of potential risk factors are measured, withdrawals and dropouts (dropout rates and completion of study rates), and no selective reporting of results.<br>In addition, this tool has four considerations to apprise domains quality:<br>1. Is there a sufficient number of events (rule of thumb, 10 events per risk factor)?<br>2. Is there sufficient presentation of data to assess the adequacy of method and analysis?<br>3. Is the strategy for model building (i.e. inclusion of variables) appropriate and based upon a conceptual framework?<br>4. Is the selected model adequate for the design?<br>Each criteria was assessed as being met (yes/no/partial/unsure). Consequently, assessment of domain criteria will assist the classification of overall study quality.<br>- **Classification of study quality**<br>The quality of the studies will be classified according to the assessment framework for assessing quality and methodological consideration in the analysis, meta-analysis and publication of observational studies [11] as high, moderate, low and very low quality based on the following criteria:<br>High: yes for all four criteria<br>Moderate: yes for criteria 1 and at least 2 other criteria<br>Low: no for criteria 1 and no or partial for 2 other criteria<br>Very low: no for criteria 1 and no or partial for all 3 other criteria.<br>- **Unit /scale of analysis issues**<br>The unit of analysis is based on the individual patient.<br>- **Assessment of heterogeneity**<br>The studies will be assessed by considering variability in the clinical and methodological heterogeneity to ensure sufficient homogeneity and rigor of the studies to conduct the meta-analysis procedure. Clinical heterogeneity of the studies will be assessed by considering their population, risk factor description and outcome. Methodological heterogeneity will be assessed using data extracted on study design,

		procedure, and analysis reported. In the absence of clinical and methodological heterogeneity statistical measures, I^2 and H^2_M statistic measures [22, 23], will also be used to determine the level of consistency between studies. I^2 and H^2_M values will be used to measure the impact of heterogeneity between and within the study variance. An $I^2 \geq 50\%$, which corresponds to $H^2_M >$ zero, will be considered a significant level of heterogeneity.<br>- **Assessment of publication bias**<br>The risk of publication bias will be minimized by comprehensively searching databases, and obtaining data from unpublished work to reduce the risk of reporting bias [24, 25]. Moreover, if more than 10 studies are reviewed that fit the criteria, a funnel plot will be used to determine the relationship between study size and effect power of cohort clinical trial studies by signs of asymmetry.
Data synthesis	15a	A meta-analysis may not be able to be conducted due to the clinical or statistical heterogeneity of the eligible studies. However, if included studies are sufficiently homogenous and rigorous, a meta-analysis using a random-effects model will be conducted. A relative risk will be the appropriate indicator of effect for potential correlates in this review with a 95% confidence interval (CI) calculated for binary outcomes (PI development). However, if there is considerable clinical and design heterogeneity in the included studies, the findings will be presented in narrative form including tables to aid in data presentation where appropriate.
	15b	The studies will be assessed by considering variability in the clinical and methodological heterogeneity to ensure sufficient homogeneity and rigor of the studies to conduct the meta-analysis procedure. Clinical heterogeneity of the studies will be assessed by considering their population, risk factor description and outcome. Methodological heterogeneity will be assessed using data extracted on study design, procedure, and analysis reported. In the absence of clinical and methodological heterogeneity, a statistical measure will also be used to determine the level of consistency between studies, I^2 statistic [19]. I^2 values will be used to measure the percentage of total variation between studies with more than 50% will considered a significant level of heterogeneity.
	15c	Nil proposed additional analyses at this stage.
	15d	Patient centred clinical risk factors for PI development will be categorized by domains and subdomains according to Coleman and colleagues [11]. Coding contributory factors into different domains will be conducted by the two authors independently. The identified domain and sub-domain factor will be summarized and presented in tables. Each sub-domain factor will be summarized be including a number of significant studies using a multivariable analysis, the total number and quality of the studies entering the variable. Disagreements will be resolved by discussion to achieve consensus or, if this is not possible, by a third independent reviewer. Given the potential considerable clinical and design heterogeneity in the included studies, quantitative synthesis may not be possible. In this case the findings will be presented in narrative form including tables to aid in data presentation where appropriate.
Meta-bias(es)	16	The quality of the body of evidence considers within-study risk of bias (methodological quality), directness of the evidence, heterogeneity of the data, precision of effect estimates and risk of publication bias.
Confidence in cumulative evidence	17	We will use the principles of the Grades of Recommendation, Assessment, Development and Evaluation (GRADE) system to assess the quality of the body of evidence associated with specific outcomes (pressure injury development) and will construct the SoF table using

	GRADE software. The GRADE approach appraises the quality of a body of evidence according to the extent to which one can be confident that an estimate of effect or association reflects the item being assessed.

*** It is strongly recommended that this checklist be read in conjunction with the PRISMA-P Explanation and Elaboration (cite when available) for important clarification on the items. Amendments to a review protocol should be tracked and dated. The copyright for PRISMA-P (including checklist) is held by the PRISMA-P Group and is distributed under a Creative Commons Attribution Licence 4.0.**

From: Shamseer L, Moher D, Clarke M, Ghersi D, Liberati A, Petticrew M, Shekelle P, Stewart L, PRISMA-P Group. Preferred reporting items for systematic review and meta-analysis protocols (PRISMA-P) 2015: elaboration and explanation. BMJ. 2015 Jan 2;349(jan02 1):g7647.
