# Supplementary figures and images for: Risk factors for pressure injury development in critically ill patients in the intensive care unit: a systematic review protocol
Source: Syst Rev. 2017 Mar 20;6:58. doi: 10.1186/s13643-017-0451-5 (PMC5359849; doi:10.1186/s13643-017-0451-5)

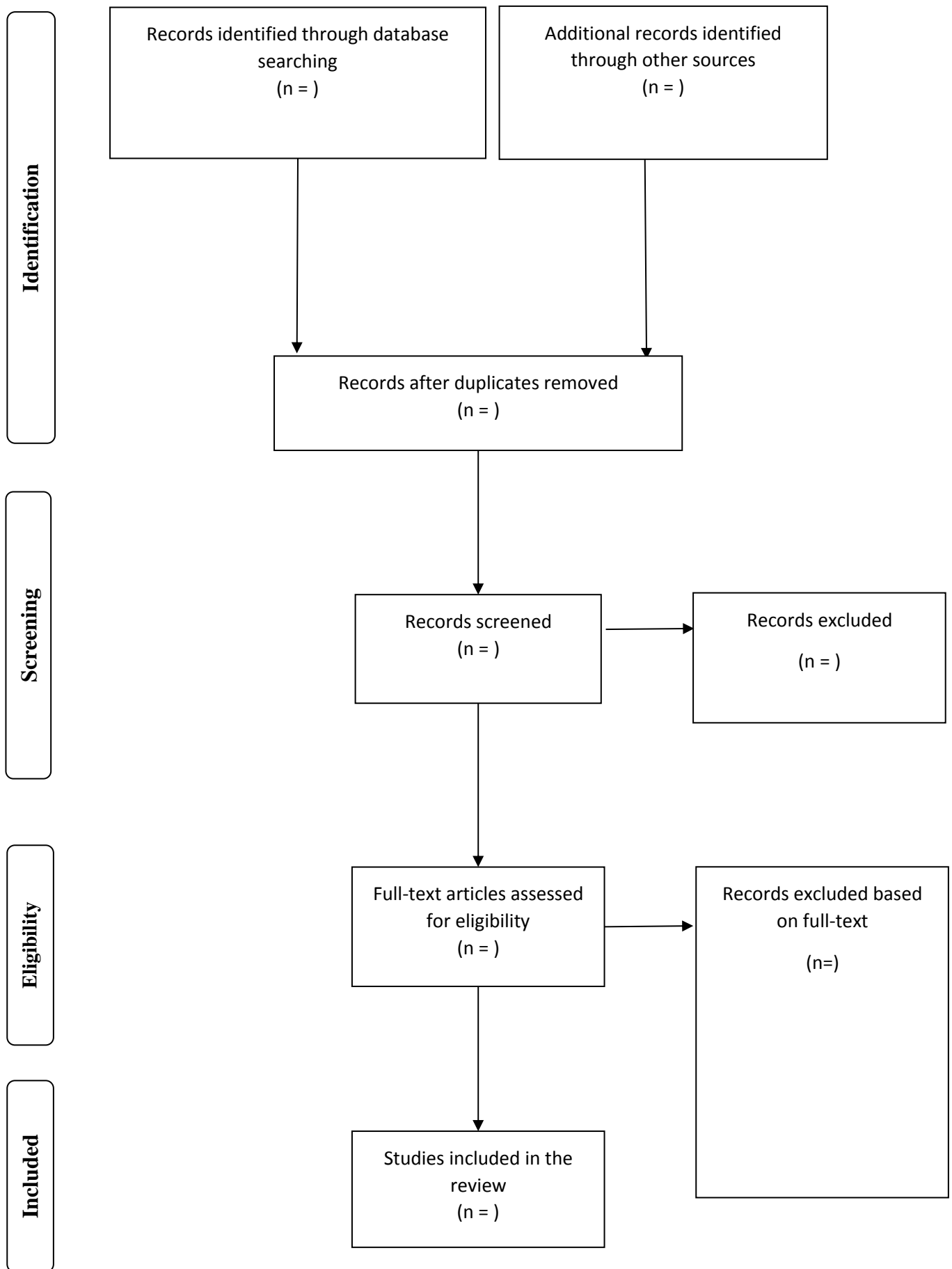

**Figure1.** Literature searches, screening and selection of articles for inclusion.

Supplement: Additional file 2: Figure S1. — Literature searches, screening and selection of articles for inclusion. (PDF 102 kb) [file 13643_2017_451_MOESM2_ESM.pdf]
